# Characteristics of the Basel Postpartum Hypertension Cohort (Basel-PPHT Cohort): An Interim Analysis

**DOI:** 10.3390/diagnostics14131347

**Published:** 2024-06-25

**Authors:** Thenral Socrates, Céline Wenker, Annina Vischer, Christina Schumacher, Fiona Pugin, Andreas Schötzau, Michael Mayr, Irene Hösli, Beatrice Mosimann, Olav Lapaire, Thilo Burkard

**Affiliations:** 1 Medical Outpatient Department and Hypertension Clinic, ESH Hypertension Centre of Excellence, University Hospital Basel, Petersgraben 4, 4031 Basel, Switzerland; celine.wenker@stud.unibas.ch (C.W.); annina.vischer@usb.ch (A.V.); christina.schumacher@usb.ch (C.S.); michael.mayr@usb.ch (M.M.); thilo.burkard@usb.ch (T.B.); 2 Eudox Statistische Beratung, 4031 Basel, Switzerland; fiona.pugin@eudox.ch (F.P.); info@eudox.ch (A.S.); 3 Department of Obstetrics and Gynecology, University Hospital Basel, 4031 Basel, Switzerland; irene.hoesli@usb.ch (I.H.); beatrice.mosimann@usb.ch (B.M.); olav.lapaire@usb.ch (O.L.); 4 Department of Cardiology, University Hospital Basel, 4031 Basel, Switzerland

**Keywords:** cardiovascular risk, high risk, maternal health, arterial hypertension, hypertensive disorders of pregnancy, diagnostics, telemedicine

## Abstract

Postpartum hypertension (PPHT) is hypertension that persists or develops after delivery and is a frequent cause of readmission, affecting 10% of pregnancies. This interim analysis aims to describe the cohort and to determine the feasibility and acceptance of a home-based telemonitoring management strategy (HBTMS) in PPHT patients. Enrollment at the University Hospital Basel began during the 2020 SARS-CoV-2 pandemic. Maternity-ward patients were screened for preexisting hypertension, hypertensive disorders of pregnancy, and de novo PPHT. In this pragmatic non-randomized prospective trial, the participants chose the HBTMS or standard of care (SOC), which consisted of outpatient hypertension clinic appointments. The HBTMS was a smartphone application or a programmed spreadsheet to report blood pressure (BP), followed by telephone consultations. Three months postpartum, the participants underwent a 24 h BP measurement and a blood, biomarker, and urine analysis. A total of 311 participants were enrolled between 06/20 and 08/23. The mean age was 34 (±5.3) years. The current pregnancy history demonstrated the following (≥1 diagnosis possible): 10% had preexisting hypertension, 27.3% gestational hypertension, 53% preeclampsia (PE), 0.3% eclampsia, 6% HELLP (hemolysis, elevated liver enzymes, and low platelets), and 18.3% de novo PPHT. A family history of cardiovascular disease and PE was reported in 49.5% and 7.5%, respectively. In total, 23.3% were high-risk for PE. A total of 68.5% delivered via c-section, the mean hospitalization was 6.3 days (±3.9), and newborn intrauterine growth restriction occurred in 21%. A total of 99% of the participants chose the HBTMS. This analysis demonstrated that the HBTMS was accepted. This is vital in the immediate postpartum period and pertinent when the exposure of hospital visits should be avoided.

## 1. Introduction

Postpartum hypertension (PPHT) is elevated blood pressure that persists or develops directly after delivery and affects 10% of all pregnancies [1]. In general, PPHT is triggered by a hypertensive disorder of pregnancy (HDP), which includes preexisting and gestational hypertension, preeclampsia, eclampsia, and HELLP (hemolysis, elevated liver enzymes, and low platelets) [2]. Although HDP are the most common trigger of PPHT, it can also be caused by transient elevated blood pressure (BP) that occurs directly after birth due to pain, volume overload, medications, or cerebral vasoconstriction syndrome, among other causes [3]. HDP accounts for 18% of maternal deaths worldwide, and with its direct link to PPHT, it should be considered with similar gravity [3,4].

However, medical guidelines focus mainly on the antenatal management of women with aHDP or women specifically with preeclampsia during pregnancy. This is despite the fact that patients can develop high BP up to 4 weeks into the postpartum period [1,3]. In addition, there is sparceare few data available about the evaluation, management, and complications of women with PPHT even though the condition of PPHT is one of the most common causes of readmission after delivery and discharge [5]. It is important to note that data regarding intermediate and long-term outcomes only exist in the context of preeclampsia, which represents only a part of the spectrum of PPHT. Although the National Institute for Health and Care Excellence (NICE) gives guidance leaning towards strict postpartum controls, there is no consensus on what is safe, feasible, and accepted, especially in the setting of managing one’s own health in terms of PPHT and the needs of a newborn baby [6]. 

In this context, we established the Basel Postpartum Hypertension Cohort (Basel-PPHT), a register enrolling all patients with PPHT and collecting follow-up data for up to 5 years. The aim of this manuscript is to describe the design of the registry, baseline characteristics, and inclusion rate after the first 2 years. Additionally, we report about the choice of the patients regarding their post-discharge management plan (remote vs. in-hospital visits) and the patients’ acceptance of a home-based telemonitoring management strategy (HBTMS). 

In the future, this cohort aims to assess the long-term cardiovascular and renal outcomes in women with PPHT, as the evidence indicates that these women have an increased long-term cardiovascular risk. Additionally, we will evaluate short-term outcomes of remote disease management options in this disease setting [7,8]. 

## 2. Materials and Methods

### 2.1. Ethics

The study protocol complies with the Declaration of Helsinki; was approved by the local ethics committee, Ethikkommission Nordwest- und Zentralschweiz (Ethics Commission Northwest and Central Switzerland) (EKNZ 2020-00736); and was registered (https://clinicaltrials.gov/ct2/show/NCT04690660, accessed on 1 May 2024). Informed consent was obtained from all of the participants.

### 2.2. Study Procedures, Design, and Definitions 

Basel-PPHT is a single-center prospective observational cohort study, currently with three nested substudies, based at the University Hospital Basel, Switzerland. 

This study takes place in close cooperation with the Hypertension Clinic of the Medical Outpatient Department and the Department of Obstetrics and Gynecology at the University Hospital Basel. Pregnant women were screened and recruited by the treating physicians in both departments and the study team. The patients were treated and follow-up occurred according to the local standards of the Hypertension Clinic of the Medical Outpatient Department. The inclusion data were collected from the hospital’s electronic clinical documentation system, from a structured interview with self-reported information at enrollment, and entered into an electronic database. The key time-points of this study were the baseline, defined as inclusion into the study with informed consent; landmark visit 1 (approximately 3 months after delivery); landmark visit 2 (approximately 6 months after delivery); and landmark 3 (approximately 12 months after delivery). 

Enrollment started in June 2020, and at the time of this interim analysis, in March 2023, the cohort consisted of 311 patients out of a planned 480 recruitments. 

All women with HDP and PPHT (defined as blood pressure measurements of systolic ≥140 and/or diastolic ≥90 mmHg or the indication of antihypertensive therapy up to six weeks after delivery) or women with preexisting hypertension or women on antihypertensive medication after delivery with an age ≥18 years were eligible [9]. As mentioned above, hypertensive disorders of pregnancy (HDP) includes preexisting and gestational hypertension, preeclampsia, eclampsia, and HELLP (hemolysis, elevated liver enzymes, and low platelets). These diseases sometimes overlap and were diagnosed by the treating obstetrician. To detect PPHT in women without antihypertensive medication, routine blood pressure measurements (BPMs) were taken in the hospital several times while monitoring vital signs during nurses’ rounds. A minimum of at least two blood pressure measurements using a Welch Allyn upper arm cuff sphygmomanometer with a BP of >139 mmHg systolic and/or >89 mmHg diastolic was required to diagnose PPHT. The exclusion criteria consist of being <18 years of age and the lack of consent to participate in the study, language barriers, or the lack of general understanding regarding the study and consent. 

The definition of high risk for preeclampsia was not based on any single criterion and was either based on the course and outcome of a previous pregnancy, decided by the treating obstetrician during the pregnancy, or based on the fetal medicine calculator cut-offs—https://fetalmedicine.org/research/assess/preeclampsia/first-trimester (accessed on 1 May 2024)—for the risk assessment of preeclampsia during pregnancy. This risk calculation tool uses Bayes theorem and combines various factors including medical history and biophysical and biochemical measurements to estimate the risk of PE in the pregnancy [10]. 

### 2.3. Blood Pressure Monitoring: In-Hospital and after Discharge 

As mentioned above, blood pressure was monitored during clinic visits with the nursing staff. This standardized measurement was taken with a Welch Allyn7000-APM (Hechingen, Germany) blood pressure device programmed to take 5 automatic unattended measurements. These measurements were then automatically transmitted to the patient’s electronic chart and reviewed by the treating physician. After discharge, the patient’s BP was monitored as clinically indicated, either with planned outpatient visits, an asynchronous telemonitoring system, or a programmed spreadsheet. During outpatient visits, an unattended automatic office blood pressure measurement was taken, and at landmark visits, an ambulatory 24 blood pressure measurement was taken. The various monitoring protocols are described below.

- Asynchronous telemonitoring:

The telemonitoring system used at the University Hospital Basel was the HEKA SMBP mobile phone application (App) (HEKA HEALTH, San Mateo, CA, USA), a customized order entry system where the physician decides when the patients should start their home blood pressure measurement period. A BPM prescription or order entry is submitted into an online portal, consisting of a period of 7 days. During the 7 days, patients are reminded via an alert on their smartphone to measure their blood pressure in the morning and in the evening, with a 12 h interval between the measurements. Each set of measurements is guided by the App, instructing the patient on how to measure their blood pressure correctly. In brief, all the patients were advised to use a clinically validated upper arm cuff device for BPMs. The patients were instructed to rest 5 min before the first measurement and 1 min between the first and the second measurement. The BP values are entered directly into the App after each measurement. After the measurement period, the physician receives the mean value of all correctly executed BP measurements. A valid measurement period was defined by the App when the patient measured their BP twice within an interval of a minute in the morning and evening for at least three days within the seven-day period. In addition, the morning and evening sets of measurements were required to be at least six hours apart for the measurement period to be considered valid and therefore generate a mean blood pressure. The HEKA App currently is only compatible with an iOS System; therefore, those patients who had the clinical indication for blood pressure monitoring after discharge but did not have an iOS system were given a programmed excel spreadsheet instead.

- Excel spreadsheet

Initially, we planned to offer remote BP measurements only to patients with an iOS smartphone to ensure correct measurements. However, due to the COVID-19 pandemic with several lockdowns, there was an urgent need of making telemonitoring and virtual BP management more accessible [11]. Therefore, we provided patients without an iOs smartphone the possibility to report their home BP with a programmed excel spreadsheet. The excel spreadsheet (Supplementary Material Document S1) was created by the research team and designed so the patient could document 7 days of measurements. Each day consisted of two measurements in the morning and two in the evening. The datasets were entered by the patient into the excel spreadsheet and then an average of the morning and evening values and of the values of the complete week was automatically calculated.

- Ambulatory 24 h Blood Pressure Measurement

For the ambulatory 24 h blood pressure measurement, a Mobil-O-Graph, Mobil-O-Graph PWA, or Spacelabs 90217A device was used [12]. The 24 h blood pressure devices were programmed to take BP measurements every 20 min from 08:00 to 22:00 and every 30 min from 22:00 to 8:00. Individual participant diaries were used to define the awake and asleep times. Their corresponding analysis software (Spacelabs Healthcare Inc., Snoqualmie, Washington, USA and Mobil-O-Graph, IEM GmBH, Aachen, Germany) analyzed the BP measurements after the 24 h BP measurement period. The mean systolic and diastolic 24 h, awake, and asleep BP values were calculated.

- Unattended automated office blood pressure measurement

The patients were placed alone in a quiet room and instructed to sit upright in a chair with uncrossed legs and both feet on the ground. The correct cuff was selected to match the arm circumference and was positioned at the level of the heart. The nurse performed a test measurement to check if the device was working properly and then started the test sequence and left the room. During the recorded measurement itself, the device automatically took three measurements after 5, 7, and 9 min. We used a Welch Allyn Connex^®^ Spot Monitor Welch Allyn7000-APM (Hechingen, Germany) that applies the SureBP^®^ measurement technique by Welch Allyn, which has been validated according to the American National Standards Institute/Association for the Advancement of Medical Instrumentation SP10:2006 (AAMI) and the British Hypertension Society (BHS) 1993 protocols [13,14]. The device was programmed to calculate the mean of these three measurements. 

### 2.4. Routine Blood and Urine Sampling 

All the clinically indicated laboratory panels were collected from the patients at the postpartum landmark visits: 3 months and, if clinically indicated, at 6 months, 12 months, and yearly thereafter. These basic blood draws were immediately processed, and the results were accessible to the treating physician and guided clinical decision-making. Additional aliquots of blood were obtained only if the patient consented to the biomarker substudy and were timed to correspond to the clinically indicated blood draws. 

### 2.5. Quality of Life and Disability 

The European Quality of Life 5 Dimensions questionnaire (EQ-5D) (Supplementary material Document S2) is a widely used, non-disease-specific preference-based instrument to measure health-related quality of life and is used for this purpose in the current study. This tool was implemented in our study at baseline to obtain a profile of patient health on the day of recruitment into the study.

### 2.6. Home-Based Telemonitoring Management Strategy and Standard In-Hospital Management 

At enrollment, the women included in the Basel-PPHT cohort with in-hospital elevated blood pressure and who had an indication for further blood pressure monitoring were informed about two possibilities of follow up. The HBTMS and standard management with in-hospital appointments after discharge. 

Those who were in the HBTMS group had scheduled telephone consultations with a treating physician from the Hypertension Clinic after SMPB periods. In general, the initial post-discharge consultation with the telemonitoring blood pressure measurement occurred one week after discharge, and the following measurement periods were determined based on clinical judgement. The number of telemedicine consultations was decided by the treating physician and was dependent on the indication for further up or down titrations of medications and blood pressure-related symptoms. Once the patients were stable in terms of blood pressure, the telemedicine visits were stopped, and all the patients underwent a 24 h blood pressure measurement three months after giving birth. If relevantly elevated blood pressure was detected 3 months after giving birth, then a repeat 24 h blood pressure measurement was performed at 6 months postpartum and yearly after that.

**Figure 1:**
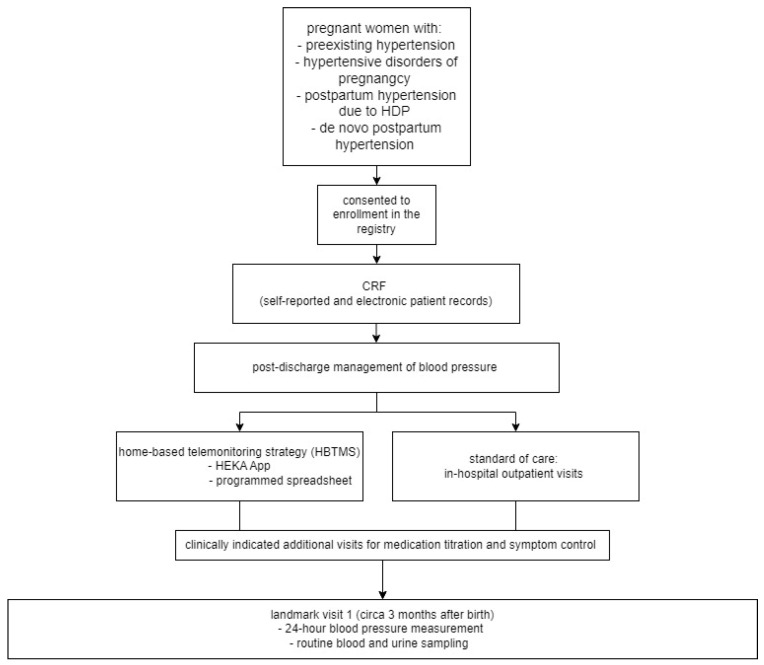
A flow chart of the Basel-PPHT cohort, including the screening, enrollment, and management after discharge.

The standard in-hospital management consisted of outpatient visits in our Hypertension Clinic 1–2 weeks after discharge and thereafter as clinically indicated (Figure 1).

### 2.7. Cardiovascular and Renal Biomarker Profiles Substudy 

An additional substudy in the Basel-PPHT cohort consists of biomarker blood banking for later analysis. This occurs at baseline (enrolment) and three, six, and twelve months after delivery. The biobanking takes place at the University Hospital Basel and the analyses are planned to be performed in batches. The serum monovettes were centrifuged immediately and aliquoted into cryotubes and stored at –80 °C at the general clinical research laboratory and Department of Biomedicine at the University Hospital Basel. The aim is to investigate the prognostic value of biomarkers for disease progression and cardiovascular events.

### 2.8. Cardiac Imaging Substudy 

In cases of persistent hypertension 6 months after delivery, a transthoracic echocardiography was routinely carried out. We plan to enroll patients into two age-matched control cohorts (Supplementary Table S1), with a sample size of 40 women per group. The first control group will consist of women with transient PPHT and normalization of blood pressure free of medication by the time of the first landmark visit (approximately 3 months postpartum). The second control group will comprise women who have given birth after an uncomplicated pregnancy. The matched control cohorts will strengthen the statistical comparison of women with persistent hypertension.

### 2.9. Questionnaires/Patient-Reported Outcome Measures (PROMs)

We evaluated the patient-reported outcome measures of the HBTMS group with a questionnaire that we developed (Supplementary Document S3). This questionnaire was completed at the three-month follow-up during the patient’s clinical visit.

#### 2.9.1. Statistical Methods 

The distribution of continuous variables will be determined using skewness, kurtosis, and visual inspection of the histograms. Continuous data will be reported as the mean ± standard deviation or median (interquartile range) and compared by means of analyses of variance (ANOVA) and Mann–Whitney U tests depending on the variable distribution. Categorical variables are described as counts (percent) and compared to chi-square tests. 

In addition to diagnostic accuracies for investigations, receiver–operator characteristic curves are calculated, as well as the sensitivities, specificities, and positive and negative prediction probabilities. Kaplan Meier and logistical regression will be used where appropriate. The correlation coefficients, variability, and standard deviation of the blood pressure measurements were used. 

#### 2.9.2. Sample Size Calculation

As this is an observational hypothesis-generating cohort study, no sample size calculation was made except for the control group of the cardiac imaging substudy. Previous studies investigating baseline to 6-month changes in echocardiographic measurements of women with persistent PPHT are currently not available. Furthermore, there is a lack of data on the new echocardiographic parameters that we are planning to study, such as the myocardial work index. We therefore have referenced the PICk-UP (Postnatal enalapril to Improve Cardiovascular fUnction following preterm Preeclampsia) study to determine our sample size calculation [15]. The PICk-UP study was a single-center randomized control trial focused on women with preterm preeclampsia, who have an 8-fold risk of death from future CVD. Women were randomized to enalapril, to improve postnatal cardiovascular function, or placebo for 6 months. Echocardiography and hemodynamic measurements were performed at baseline (<3 days), 6 weeks, and 6 months post-delivery on 60 women. We hypothesize that 25% of our participants will have concentric remodeling after 6 months of persistent hypertension and that, of the control group consisting of healthy women without hypertension, less than 3% will present with concentric remodeling. Therefore, with an alpha value of significance of 0.05 and an 80% power to detect an effect size, we need 40 healthy controls. We plan to recalculate our sample size calculation after the inclusion of 40 participants with persistent hypertension at 6 months. 

The statistical analysis was performed using statistical software R (version 4.2.1), and a *p*-value of <0.05 is pre-specified to indicate statistical significance.

## 3. Results

### 3.1. Inclusion Trends 

**Figure 2:**
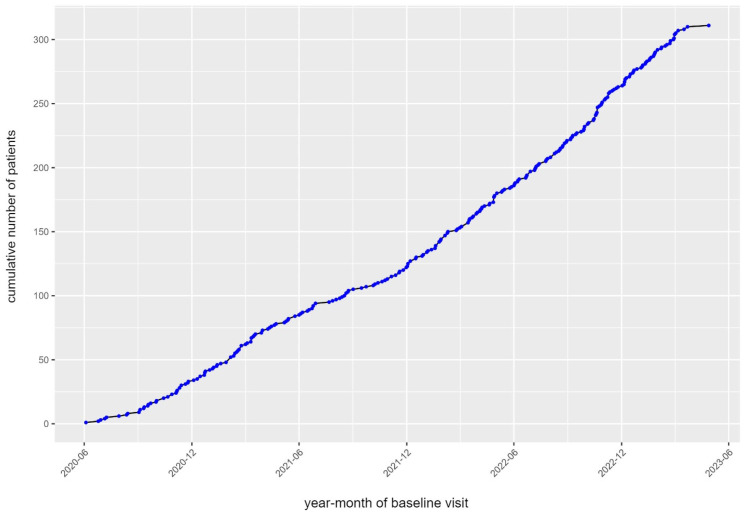
Inclusion into the registry starting in June 2020.

Up to March 2023, we included 311 patients in the registry. Inclusion into the registry, depicted in Figure 2, started in June 2020, at the beginning of the COVID-19 pandemic, and rapidly increased over the course of 2021–2022. 

### 3.2. Baseline Characteristics 

Table 1 shows the baseline characteristics of the cohort. The mean (±standard deviation) age was 33.8 (5.3) years. The average BMI was 27 (6.2) kg/m^2^. The mean weight before pregnancy was 71 kg (±18) and after delivery 83 (±20) kg. A total of 84.8% of the cohort were Caucasian, 4.6% were Asian, 6% were Black or African Origin, and 2.7% were Middle Eastern. In total, 20% had a university level education, 17.3% a secondary university degree, and 55.3% vocational training. A total of 64.3% of the women self-reported a healthy diet without regular intake of increased caffeine (more than two servings per day), processed foods, increased salt, or energy drinks. In total, 23.3% of the cohort was determined as high risk for preeclampsia and 29% took aspirin during the current pregnancy. The most prevalent comorbidity during pregnancy was a history of hypertension in 11.9%. A total of 10.6% had a thyroid-related disease at baseline.

Nulliparity war was seen in 22.3% of the cohort. In terms of the medical history of previous pregnancies, 30% of the cohort had one or more miscarriages prior to their current pregnancy. A total of 8% had preeclampsia in a previous pregnancy, and 61% were unsure if they had a complication in their last pregnancy. 

### 3.3. Cardiovascular Risk Factors

The most common self-reported cardiovascular risk factor was a positive family history of cardiovascular disease in 49.5% of the women. In total, 21.2% had smoked, currently smoked, or smoked occasionally; of these, 6.1% were still active. A history of exposure to pollution, city dwelling, and occupational exposure combined was reported in 62% of the cohort. A positive family history of preeclampsia was reported in 7.5%. Table 2 provides information about different cardiovascular risk factors at baseline. 

### 3.4. Hypertension and Relevant Medical History of the Current Pregnancy 

Preeclampsia was present in 53% of the cases. Gestational hypertension occurred in 27.3% and was the second most prevalent condition, followed by isolated postpartum hypertension at 18.3%. Table 3 depicts the relevant hypertensive and medical history of the current pregnancy.

### 3.5. Delivery Characteristics

Table 4 summarizes the types of deliveries of the patients in the cohort. The majority of women, 68.5%, delivered by cesarean section; 23% had a vaginal birth; and 8.4% gave birth by vacuum extraction. The average week of gestation at delivery was 36.8 ± 3.3. Intrauterine growth restriction was diagnosed in 21% of the newborns, with 12.4% of those being born before 37 weeks of gestation. The average birth weight of the newborns was 2828 (±863.3) grams.

### 3.6. Baseline Medication

**Figure 3:**
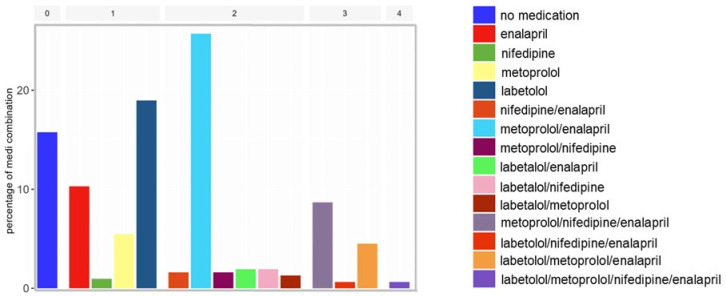
Type and percentages of baseline antihypertensive medications. Combination of medications: 0–4 medication combinations; color legend on the right with the various combination of medications.

At baseline, 86.4% (266), missing (*n* = 3), of the patients were taking antihypertensive medication. Monotherapy was present at baseline in 35.7% (111) and more than one medication in 48.6% (151). The following medication distribution was documented: 30% (93) labetalol, 48% (149) metoprolol, 16.1% (50) nifedipine, and 54% (168) enalapril. The various combinations of medication are shown in Figure 3. 

### 3.7. Blood Pressure Values 

The mean maximum systolic and diastolic blood pressure was 168 ± 17.1 and 106 mmHg ± 18.1, respectively. The mean maximum heart rate was 115± 29 beats per minute (bpm). Table 5 shows, in addition to the mean (SD), the median and minimum and maximum blood pressure values. 

### 3.8. Patient-Chosen Management Plan

Table 6 depicts patient chosen post-discharge management, 99% of the women preferred a home-based telemonitoring management plan. A total of 65% used the telemonitoring App to document their blood pressure. 

## 4. Discussion

The Basel PPHT Register comprises a cohort of technologically perceptive women who gave birth during a pandemic. Although the conception of this study occurred in 2019 before we could have imagined a global SARS-CoV-2 pandemic, this event acted as a catalyst for the hospital to quickly accept and opportunely implement the idea of a home-based management strategy. In general, a positive aspect of the pandemic was that it pushed telemonitoring further and faster than anyone could have imagined. The pandemic instigated the acceptance of patients to stay out of the hospital and embrace a new health care management modality.

We showed that in Switzerland, PPHT affects, on average, 34 year olds, which is slightly older than the average age of giving birth in Switzerland, which lies currently at 30.7 (https://www.bfs.admin.ch/bfs/en/home/statistics/population/births-deaths.html accessed on 01.05.2024). This is in line with data that show that in general women with preeclampsia are either younger or older than average women in terms of maternal age at birth [16]. The PPHT Registry mainly consisted of Caucasian women, followed by Black or African Origin 6% and 4.6% Asian, which is high considering the Swiss population has 8% permanent Asian residents (https://www.bfs.admin.ch/bfs/de/home/statistiken/bevoelkerung/migration-integration/auslaendische-bevoelkerung.assetdetail.26565228.html accessed on 1 May 2024). BMI before pregnancy was slightly over the normal range at 27 kg/m^2^. 

Interestingly, although the majority of this cohort had preeclampsia at 53%, gestational hypertension was seen in 27.3% and de novo PPHT in 18.3%. This is important and differentiates this cohort to others because generally the focus in postnatal care is focused on patients with preeclampsia or eclampsia. Importantly, as demonstrated in this cohort, PE only accounts for just over half of the population with elevated BP values after birth. 

A positive family history of cardiovascular disease was seen in about half of the cohort. This is of interest as this was self-reported and could be used in the future to help screen for women at an increased risk of PPHT and bring awareness regarding elevated blood pressure in the postpartum period [16]. Furthermore, HDP, the most common cause of PPHT, impacts not only a woman’s pregnancy but also her offspring. The data show that HDPs are an epigenetic trigger for future cardiovascular disease in exposed offspring; this may help in engaging patients to take more action regarding strict blood pressure controls, adherence, and lifestyle choices during their pregnancy [17]. 

In this population, 23% of the women in our cohort were already determined to be high risk for preeclampsia during the current pregnancy and 29% were taking aspirin. This is especially interesting when we considered that 51% of the cohort had preeclampsia. This implies that with the actual and local risk screening methods, not all patients are identified. 

Almost two-quarters of the cohort had a cesarean section, which is associated with increased complications compared to vaginal birth for the current pregnancy and future pregnancies [18]. The average hospital stay of the cohort was 6.3 days. 

The birth weight on average was 2828 g. A total of 21% of the newborns had intrauterine growth restriction and 12.4% were born before 37 weeks of gestation. These characteristics are cardiovascular risk factors for the offspring later in life [19]. This aspect of HDP and PPHT is again associated with poor outcomes [20]. 

Most women were taking an antihypertensive medication at baseline. According to guidelines, a switch to longer acting and more beneficial medications such as enalapril should be made after giving birth. In most cases, we switched the patients to metoprolol and enalapril with nifedipine as a third-line or reserve medication after delivery. Enalapril is especially justified because of its cardiac- and renal-protective properties [15]. However, it should be noted that usually the focus of the management of PPHT is directed at women with preeclampsia or eclampsia and not with other causes of PPHT, such as chronic hypertension or de novo PPHT. The benefit of a more customized therapy strategy based on the various causes needs to be looked at in future studies. 

After birth, blood pressure is dynamic and can rapidly increase or decrease [21]. With 99% of the cohort having an indication for antihypertensive medication after delivery, it is clear that a structured and supervised titration of medication after discharge should be carried out in women with postpartum hypertension. This is in line with the NICE guidelines that recommend strict controls in the postpartum period [6]. Within our management model, we usually recommend to start taking blood pressure measurements directly after hospital discharge.

With the nested substudy on the HBTMS versus usual care, we aim to investigate how different care models are accepted and their impact on outcome. The interim data analysis showed that the home-based management model we offered was highly accepted in this patient group. The question remains whether home-based management results in better and safer outcomes in general. In 2022, the European Society of Hypertension published a consensus document regarding the virtual management of hypertension and lessons learned from the pandemic. This consensus document provides guidance on structures/SOP specifications for BP measurement, data transmission, communication with the patient, and follow-up [11]. In our cohort, we implemented many similar strategies. We are still a long way from complete virtual management; however, the immediate post-hospital setting is very suitable for a hybrid approach in this group of patients. The follow-up of our registry will answer the questions about feasibility, adherence to a home-based management approach, and efficacy and safety.

The nested substudy on the home-based telemonitoring strategy versus usual care was originally meant to be technically randomized based on having an iPhone, as the telemonitoring App that was implemented only works on an iOS System. However, in light of the SARS-CoV-2 pandemic, we reevaluated the necessity and importance of allowing all participants to choose their management strategy, especiallybecause routine care capacities were highly reduced during the lockdown periods. 

Previous studies on pregnancy-related hypertension focused mainly on preeclampsia-associated outcomes. With the Basel-PPHT cohort, we aim to contribute to closing potential gaps regarding PPHT and HDP by investigating all entities behind PPHT/HDP and their consequences for the general and cardiovascular health of affected women. Specially, in nested substudies, we aim to explore biomarkers as prognostic factors in PPHT/HDP and the impact of PPHT/HDP on heart disease.

## 5. Conclusions

The Basel PPHT Registry describes a heterogenous population of women with hypertensive disorders in pregnancy and de novo hypertension. Interestingly, most studies focusing on hypertension during and after pregnancy consist of mainly preeclamptic patients; however, about half of our cohort had another cause of PPHT. This is important, as these women, who may be considered low risk, also need adequate follow-up after delivery. We were also able to demonstrate that a management strategy using telemedicine was well received by this cohort of women. 

## Figures and Tables

**Table 1:** Baseline characteristics, *n* = 311.

age—years, mean (SD), median (IQR)	33.8 (5.3) 34 (7)
ethnicity (*n*) (%) (missing *n* = 9)	
- Caucasian	256 (84.8)
- Black or African Origin	18 (6)
- Asian	14 (4.6)
- Middle Eastern	6 (2.65)
- more than one ethnicity	2 (0.99)
- unknown	1 (0.1)
height (in cm), mean (SD), median (IQR) (missing *n* = 3)	165 (5.9) 165 (9)
weight before pregnancy (in kg), mean (SD), median (IQR) (missing *n* = 23)	71 (18) 66 (21)
weight at baseline (in kg), mean (SD), median (IQR) (missing *n* = 14)	83 (20) 79 (22)
BMI before pregnancy (kg/m^2^), mean (SD), median (IQR) (missing *n* = 14)	27 (6.2) 24 (8)
education *n* (%) (missing *n* = 16)	
- high school	10 (3.4)
- vocational/technical school	163 (55.3)
- university	59 (20)
- secondary university degree (masters, PhD)	(51) (17.3)
nutrition, % (*n*) *	
- caffeine (>2 servings per day)	18 (5.8)
- extra salt	51 (16.4)
- processed foods	26 (8.4)
- energy drinks	3 (1)
- none of the above	200 (64.3)
determined as high risk for preeclampsia between 11 and 14 weeks gestation, *n* (%)	70 (23.3)
received aspirin due to high risk for preeclampsia, *n* (%)	88 (29)
comorbidities, *n* (%) *	
- none	189 (61)
- history of hypertension	37 (12)
- cardiovascular disease	6 (1.9)
- renal disease	4 (1.3)
- thyroid disease	33 (10.6)
- sleep apnea	1 (0.3)
- arterial malformation	2 (0.6)
- diabetes	36 (11.6)
- other	28 (9)
nulliparous, *n* (%) (missing *n* = 1)	
- yes	69 (22.3)
- no	241(77.7)
medical history of previous pregnancies, *n* (%) *	
- uncomplicated	56 (18)
- gestational hypertension	12 (3.9)
- preeclampsia	25 (8.04)
- postpartum hypertension	9 (2.9)
- gestational diabetes	11 (3.6)
- previous miscarriage	60 (30)

The data are presented as the mean (±SD), median (IQR), and *n* (%) self-reported by the patients or taken from records. * more than one choice possible.

**Table 2:** Cardiovascular risk factors *.

lack of exercise, *n* (%)	59 (19)
obesity before pregnancy, *n* (%) (BMI before pregnancy >30 kg/m^2^)	61 (20)
arterial hypertension *n* (%)	35 (11.3)
dyslipidemia, *n* (%)	5 (1.6)
family history of cardiovascular disease, *n* (%)	154 (49.5)
diabetes, *n* (%)	4 (1.3)
none, *n* (%)	59 (19)
history of smoking, *n* (%)	66 (21.2)
current smoking status, *n* (%) (missing *n* = 14)	
- active	18 (6.1)
- never	183 (62)
- quit	96 (32.3)
history of exposure to pollution, *n* (%) (missing *n* = 3)	
- none	118 (38.3)
- city dwelling	179 (55.2)
- occupational exposure	20 (6.5)
family history related to hypertensive disorders in pregnancy (first degree), *n* (%) (missing *n* = 17)	
- family history of pregnancy-induced hypertension	15 (5.1)
- family history of preeclampsia	22 (7.5)
- family history of postpartum hypertension	8 (2.7)
- unknown	112 (38.1)
- none	137 (47)

The data are presented as *n* (%), self-reported by the patients or taken from records; * more than one choice possible.

**Table 3:** Course of pregnancy *.

uncomplicated until after birth, *n* (%)	57 (18.3)
preexisting hypertension, *n* (%) (<20 weeks of gestation)	30 (10)
gestational hypertension, *n* (%) (>20 weeks of gestation)	85 (27.3)
preeclampsia, *n* (%)	164 (53)
eclampsia, *n* (%)	1 (0.3)
HELLP, *n* (%)	18 (6)

Data are presented as *n* (%); HELLP = hemolysis, elevated liver enzymes, and low platelets; and * more than one disease possible.

**Table 4:** Hospital stay, type of delivery, and newborn characteristics.

number of nights in hospital, mean (SD), median (IQR) (missing *n* = 9)	6.3 (3.9) 5 (3)
- c-section, *n* (%)	213 (68.5)
- vaginal, *n* (%)	71 (22.8)
- vaginal birth after c-section (VBAC), *n* (%)	0 (0)
- vacuum, *n* (%)	26 (8)
- forceps, *n* (%)	1 (0.3)
- week of gestation at birth, mean (SD)	36.8 (3.5)
- intrauterine growth restriction, *n* (%) - (missing *n* = 5)	64 (21)
- <37 weeks of gestation	38 (12.4)
- >37 weeks of gestation	26 (8.5)
- weight of newborn in grams at birth, mean (SD)	2828 (863.3)

Data are presented as the mean (±SD), median (IQR), and *n* (%); VBAC = vaginal birth after cesarean section weight in grams.

**Table 5:** Baseline blood pressure characteristics.

max. systolic blood pressure (mmHg)	
mean (SD)	168 (17)
median (IQR)	166 (18)
minimum and maximum	126, 248
max diastolic blood pressure (mmHg)	
mean (SD)	107 (19)
median (IQR)	104 (15)
minimum and maximum	74, 230
max heart rate (bpm)	
mean (SD)	115 (29)
median (IQR)	108 (27)
minimum and maximum	61, 261

Data are presented as the mean (±SD), median (IQR), and minimum and maximum blood pressure values (missing *n* = 10).

**Table 6:** Patient-favored management plan.

Patient-favored management plan, *n* (%) (missing *n* = 6)	
- home-based	302 (99)
- standard of care	3 (1)
type of HBTMS, *n* (%) (missing *n* = 7)	
- telemonitoring App	198 (65)
- programmed spreadsheet	106 (35)

Data are presented as *n* (%).

## Data Availability

The datasets generated during and/or analyzed during the current study are not publicly available; however, they are available from the corresponding author on reasonable request.

## Supplementary Materials

The following supporting information can be downloaded at: https://www.mdpi.com/article/10.3390/diagnostics14131347/s1, Document S1: Excel Spreadsheet; Document S2: EQ-5D; Document S3: PPHT Registry Questionnaire for APP. Document S4: Supplementary Table S1.
