# Supplementary material for: Characteristics of the Basel Postpartum Hypertension Cohort (Basel-PPHT Cohort): An Interim Analysis
Source: Diagnostics (Basel). 2024 Jun 25;14(13):1347. doi: 10.3390/diagnostics14131347 (PMC11240531; doi:10.3390/diagnostics14131347)
Supplement: Supplementary file 1 [file diagnostics-14-01347-s001.zip › supplementary materials/S3.pdf]

PPHT Registry Questionnaire For App Based Monitoring

Please read the questionnaire carefully, take your time and answer honestly. Thank you !

PPHT Questionaire for App Based Monitoring

How satisfied are you with the use of the<br><br>Telemonitoring?	◯ very happy ◯ satisfied<br>◯ neutral ◯ dissatisfied<br>◯ very unhappy
Did you have any problems with the telemonitoring ?	◯ I had no issues with the App<br>◯ using the app was confusing<br>◯ the app was not visually appealing<br>◯ technical issues<br>◯ I didn't have time to use the App<br>◯ comment
what did you like most about the mobile app ?	◯ functionality<br>◯ that i did not need to come into the hospital<br>◯ that the doctor had my blood pressure measurements<br>◯ that the App lead me through the measurement process
did you feel safe using the app/home based monitoring	◯ Yes<br>◯ No
would you use the App in the future for blood pressure related issues	◯ Yes<br>◯ No
was the experience of taking part in a home based monitoring study good	◯ Yes<br>◯ No
DATE of filling out this questionnaire	________________________

You may also use DESCRIPTIVE TEXT to provide informational text within a survey section.
