# Supplementary material for: Characteristics of the Basel Postpartum Hypertension Cohort (Basel-PPHT Cohort): An Interim Analysis
Source: Diagnostics (Basel). 2024 Jun 25;14(13):1347. doi: 10.3390/diagnostics14131347 (PMC11240531; doi:10.3390/diagnostics14131347)
Supplement: Supplementary file 1 [file diagnostics-14-01347-s001.zip › supplementary materials/S4.pdf]

Supplementary Table S1. Cardiac Imaging Control Cohort.

time (months)	0<br>Control Cohort of the Cardiac Imaging Cohort<br><br>group 1: women without hypertension	+3-6 Month postpartum<br><br>Control Cohort of the Cardiac Imaging Cohort<br><br>group 2: postpartum normalization of blood pressure
visit	Screening	1st /screening for cardiac control group imaging
oral and written Information for TTE	+	+
written consent	+	+
check inclusion-/ exclusion criteria	+	+
